# Comparative evaluation of short vs. long needles in deep ultrasound-guided pharmacopuncture for acute low back pain: study protocol

**DOI:** 10.3389/fmed.2026.1822731

**Published:** 2026-06-24

**Authors:** Kwangho Kim, Young-Ung Lee, Robin Kwon, Hongmin Chu, Seongjun Park, Hyeon Joon Hong, Youngyun Lee, Junhui Kwon, Juhwan Song, Sanghyuk Kwon, Jungtae Leem, Cheol-Hyun Kim

**Affiliations:** 1 The Academy of Korean Medicine Clinical Anatomy, Seoul, Republic of Korea; 2 Gangdong Forest Hospital of Korean Medicine, Seoul, Republic of Korea; 3 Mapo Hongik Korean Medicine Clinic, Seoul, Republic of Korea; 4 Korean Medicine Convergence Research Information Center for Stroke, College of Korean Medicine, Wonkwang University, Gwangju, Republic of Korea; 5 Mullae Majubom Korean Medicine Clinic, Seoul, Republic of Korea; 6 Gwanghwamun Kyunghee Korean Medicine Clinic, Seoul, Republic of Korea; 7 Kangnyung Korean Medicine Clinic, Anyang, Republic of Korea; 8 Anjung Korean Medicine Clinic, Seoul, Republic of Korea; 9 Department of Korean Medicine, Jinjeop Hanyang Hospital, Namyangju, Republic of Korea; 10 Research Center of Traditional Korean Medicine, College of Korean Medicine, Wonkwang University, Iksan, Republic of Korea; 11 Department of Il-won Integrated Medicine, Wonkwang University Korean Medicine Hospital, Iksan, Republic of Korea; 12 Department of Diagnostics, College of Korean Medicine, Wonkwang University, Iksan, Republic of Korea; 13 Department of Internal Medicine, College of Korean Medicine, Wonkwang University, Iksan, Republic of Korea

**Keywords:** injection, low back pain, musculoskeletal pain, pharmacopuncture, ultrasonography

## Abstract

**Background/Objectives:**

Acute Low Back Pain (LBP) commonly shows rapid symptom improvement during the early phase; however, delayed or inadequate treatment may increase the risk of recurrence and chronicity. Ultrasound-guided pharmacopuncture has demonstrated promising analgesic effects, yet the optimal needle length for deep interfascial targeting remains unclear, particularly in procedures involving the Quadratus Lumborum–Psoas–Iliocostalis (QPI) space. This study aims to compare the short-term clinical response, procedural feasibility, and safety of short versus long needles used for QPI-guided pharmacopuncture in patients with acute LBP.

**Methods:**

This prospective, multicenter, comparative observational study includes adults aged 20–65 years presenting with acute LBP within 7 days of onset. Participants undergo a single session of ultrasound-guided Polydeoxyribonucleotide (PDRN) pharmacopuncture targeting the QPI interfascial plane using either a short needle (26G × 60 mm) or a long needle (25G × 100 mm). Group assignment is based on pre-designated site-level data extraction rather than randomization. The primary outcome is the change in the Numeric Rating Scale (NRS) score immediately after treatment. Secondary outcomes include changes in lumbar range of motion (ROM), patient-reported percentage pain reduction (PRPPR), and procedural usability. Safety is monitored immediately post-procedure and again during a telephone follow-up 24–36 h later. Statistical analyses will compare between-group effects and explore potential associations with procedural characteristics.

**Discussion and conclusion:**

This study aims to provide preliminary feasibility evidence on whether needle length affects procedural performance and immediate analgesic response during ultrasound-guided interfascial pharmacopuncture for acute LBP. By evaluating both clinical and procedural metrics, the results may contribute to establishing standardized needle-selection criteria and support future randomized controlled trials designed to optimize deep pharmacopuncture techniques within Korean medicine primary care settings.

**Clinical trial registration:**

https://cris.nih.go.kr/cris/search/detailSearch.do?seq=32507&search_page=L&search_lang=&class_yn=.

## 1 Introduction

Low back pain (LBP) is one of the most prevalent musculoskeletal disorders worldwide and is a major contributor to reduced quality of life and increased socioeconomic burden. In Korea, the number of patients seeking care for LBP has continued to rise, along with the growing demand for Korean medicine-based treatments. According to the *2023 Yearbook of Korean Medicine,* published in 2025 (1), the two conditions accounting for the highest proportion of outpatient expenditures in Korean medicine institutions in 2023 were back pain and lumbar sprain, underscoring the high clinical demand for LBP-related care.

The number of patients seeking care for LBP and lumbar sprain at both Korean medicine and conventional medical institutions has remained consistently high in Korea (2). These figures indicate that a substantial proportion of patients with LBP utilize Korean medicine services, highlighting the need for further standardization of interventional procedures in Korean medicine clinical practice.

Acute LBP typically shows rapid improvement within the first 6 weeks; however, recovery significantly slows thereafter (3). Despite initial improvement, symptoms often fail to resolve completely, and approximately 32% of patients with acute LBP progress to chronic LBP within 6 months (4). This transition to chronic phase is associated with higher recurrence rates, reduced quality of life, increased healthcare utilization, and substantial societal costs. Therefore, accurate assessment and timely intervention during the acute phase are critical for favorable long-term outcomes, as insufficient management of early pain increases the risk of recurrent exacerbations and chronicity.

Pharmacopuncture, a Korean medicine intervention that combines acupuncture with bioactive pharmacological agents, has demonstrated rapid analgesic effects in acute LBP, lumbar sprain, and myofascial pain syndrome. Various conservative interventions for LBP, including pulsed electromagnetic field therapy, have also been investigated for pain reduction and functional improvement in previous studies (5). Recently, ultrasound-guided pharmacopuncture has gained attention due to its ability to deliver agents precisely to the targeted anatomical structures while avoiding major nerves and blood vessels (6, 7). A recent systematic review reported that ultrasound-guided pharmacopuncture provides superior pain relief and functional improvement compared with non–image-guided procedures, including an additional 27% improvement in pain outcomes in subgroup analyses focusing on lumbar conditions (8).

However, deep lumbar interfascial structures—such as those surrounding the Quadratus Lumborum(QL) and Psoas Major(PM)—present significant technical challenges due to anatomical variability, soft tissue thickness, and reduced needle-tip visibility at greater depth (9). Conventional 60-mm needles may be insufficient for reliably reaching deep target layers, whereas longer needles (e.g., 100 mm) can provide stable access but have not been widely adopted in Korean medicine due to limited evidence regarding their safety and handling characteristics. Furthermore, the physical limitations of ultrasound, particularly reduced echogenicity at greater depth, have led to increasing clinical demand for needle-tracking technology to enhance procedural accuracy (10).

Our research team previously performed ultrasound-guided pharmacopuncture targeting the interfascial space between the QL, PM, and Iliocostalis(IC) muscles—referred to as the “QPI space”—in five patients with acute lumbar myofascial pain. This procedure resulted in significant reductions in pain and improvements in lumbar Range of Motion(ROM), with successful deep-plane access and no adverse events (7). These findings provide preliminary feasibility evidence supporting the need for systematic evaluation of needle length and procedural performance in ultrasound-guided deep pharmacopuncture.

Based on these clinical needs and previous findings, the present study aims to compare the immediate analgesic effects of short versus long needles in ultrasound-guided pharmacopuncture targeting the QPI space through a prospective, multicenter observational design. The primary outcome is the change in pain intensity immediately after treatment, enabling evaluation of how needle length influences the acute-phase clinical response. Additionally, secondary procedural and exploratory metrics—including deep-plane reachability, technical usability, and safety—will be assessed to explore the feasibility and potential for standardizing deep ultrasound-guided pharmacopuncture in primary care Korean medicine settings.

## 2 Materials and methods

### 2.1 Study design

**Figure 1:**
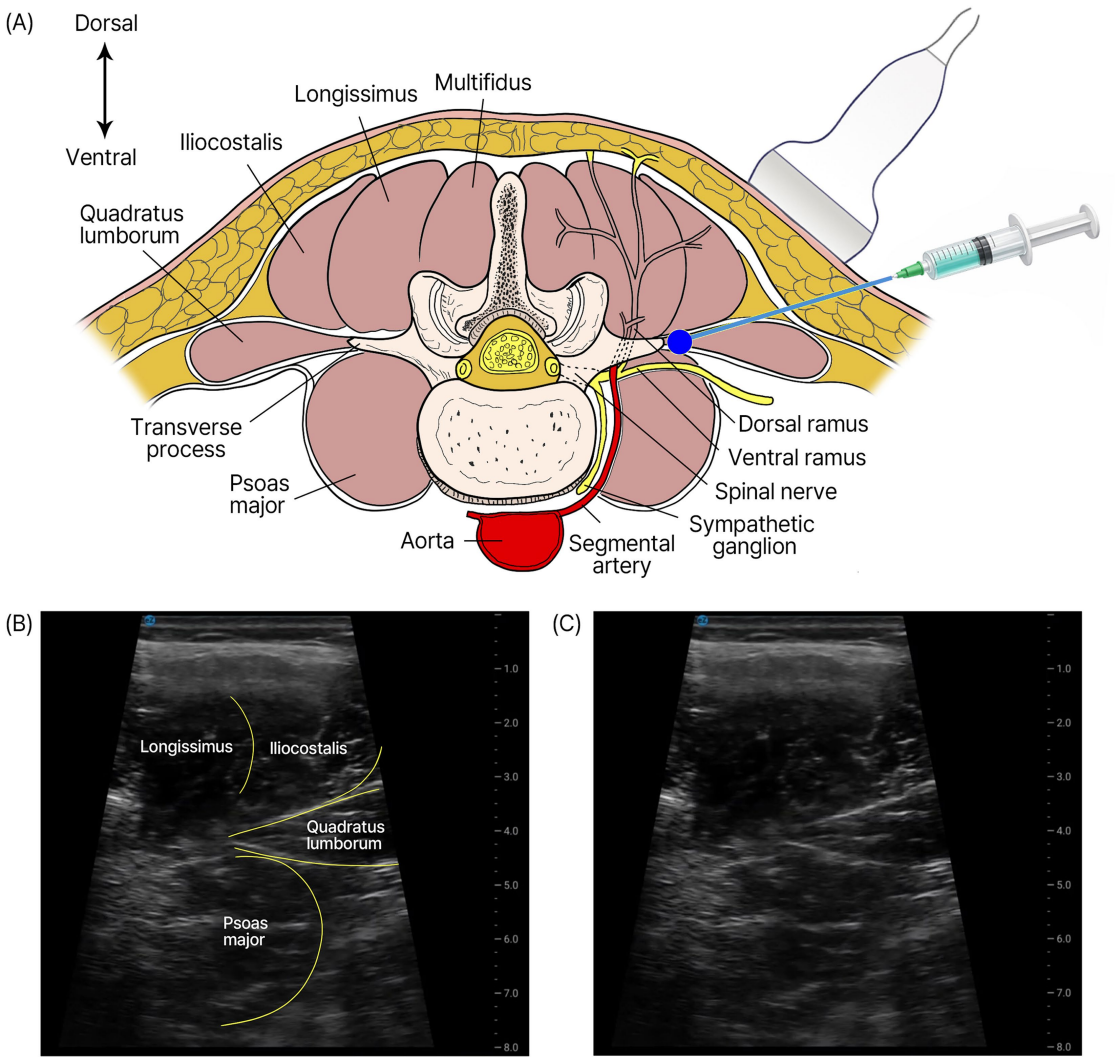
Anatomical structure and ultrasound visualization of the quadratus lumborum–psoas–iliocostalis (QPI) interfascial space. **(A)** Cross-sectional anatomi-cal illustration at the lumbar level demonstrating the anatomical relationship among the quadratus lumborum, psoas major, and iliocostalis muscles. The interfascial plane formed by these muscles, referred to as the QPI space (blue dot), and the intended needle trajectory toward the target layer are illustrated. **(B)** Representative ultrasound image with annotated muscle boundaries corresponding to the anatomical structure, showing the iliocostalis, quadratus lumborum, and psoas major muscles and the intervening QPI interfascial space. **(C)** Corresponding unannotated ultrasound image of the same scanning plane, demonstrating the native sonographic appearance of the QPI interfascial space.

This prospective, multicenter, comparative observational study aims to evaluate whether needle length (Long versus Short) influences procedural usability, deep target achievement rate, clinical effectiveness, and safety during ultrasound-guided pharmacopuncture for acute LBP. The intervention targets the interfascial plane formed by the QL, PM, and IC muscles—referred to as the “QPI space.” The anatomical location and ultrasound appearance of the QPI interfascial space are illustrated in Figure 1. All procedures are performed under real-time ultrasound guidance. Polydeoxyribonucleotide (PDRN) derived from salmon is used as the pharmacological agent, and a magnetic needle–guided tracking system is applied to deliver 1–2 mL of the injectate into the target layer.

**Figure 2:**
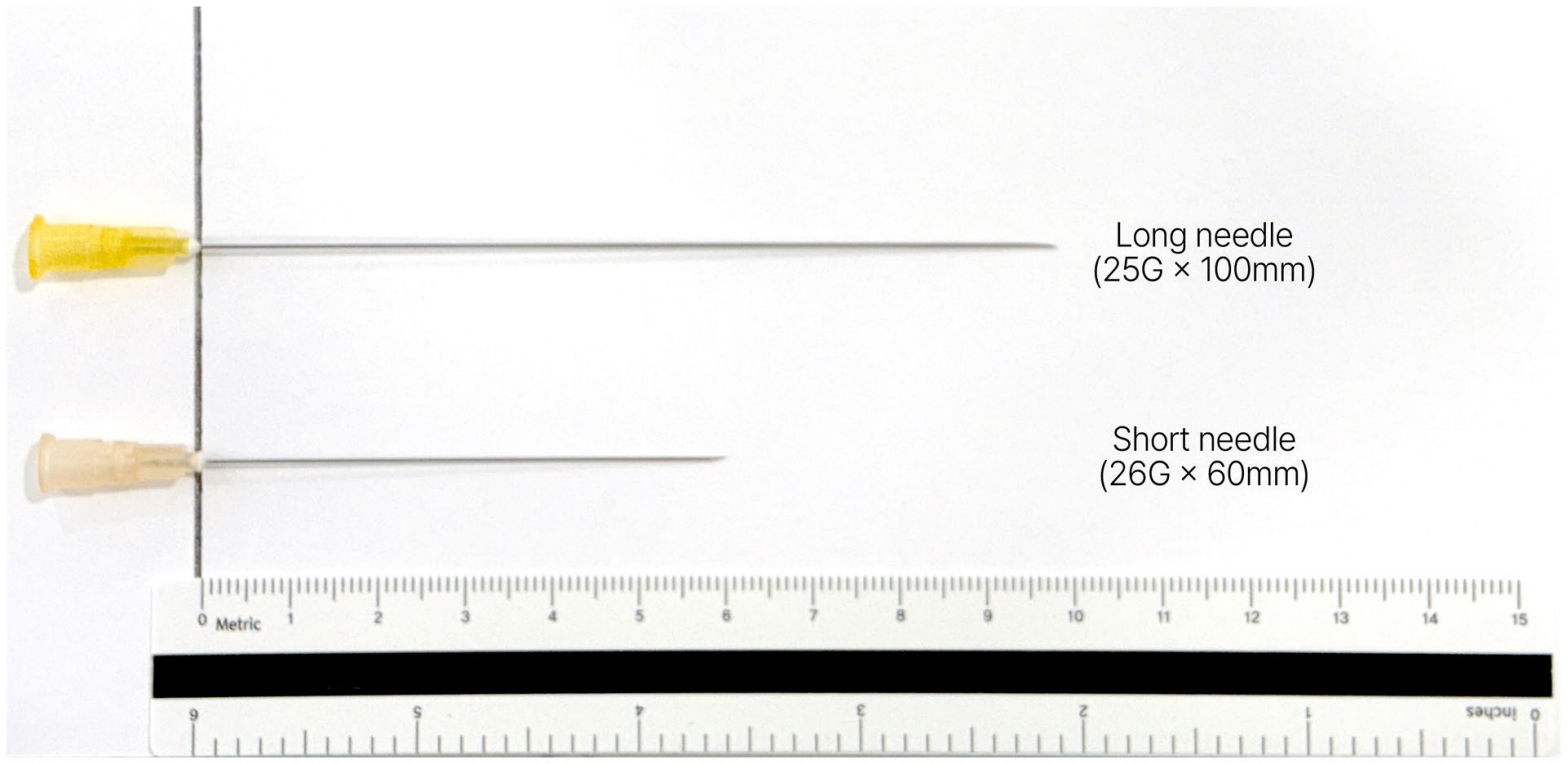
Comparison of the two magnetized needle types used in this study (26G × 60 mm and 25G × 100 mm).

Participants who express willingness to join the study will be screened according to the inclusion and exclusion criteria after receiving a full explanation of the study’s purpose and procedures and providing written informed consent. Eligible participants will be allocated to either the Short Needle Group (SNG; 26G × 60 mm) or the Long Needle Group (LNG; 25G × 100 mm). The two needle types used in this study are shown in Figure 2.

All four participating clinical sites routinely use both types of needles in daily practice, depending on actual clinical circumstances. For the purpose of comparative analysis, two sites will be designated to contribute only cases treated with short needles, while the other two sites will contribute only cases treated with long needles. This approach represents a site-level data extraction scheme, rather than a randomized allocation process, and is designed to enable comparative analysis of procedures performed in routine clinical practice without modification of standard patient care.

The study procedures consist of three stages: screening, the treatment visit (Visit 1), and post-procedure follow-up at 24 to 36 h. After providing written informed consent, participants undergo screening, during which demographic characteristics and relevant medical history are collected, and eligibility is assessed based on predefined inclusion and exclusion criteria. Participants who meet all eligibility criteria proceed directly to Visit 1, during which the ultrasound-guided pharmacopuncture intervention and all corresponding study assessments are conducted.

During Visit 1, all enrolled participants undergo a single ultrasound-guided PDRN pharmacopuncture procedure following a standardized protocol. Prior to the intervention, baseline assessments are conducted, including Numerical Rating Scale (NRS) scores for LBP and radiating leg pain, lumbar ROM, the Oswestry Disability Index (ODI), and the Patient-reported Percentage Pain Reduction(PRPPR) score. After the baseline evaluation, the practitioner identifies the interfascial QPI space using high-resolution ultrasonography and then performs the injection using a magnetically guided needle system, administering 1–2 mL of the PDRN pharmacopuncture solution.

The procedure is performed using an in-plane needle approach, enabling real-time visualization of the needle trajectory and tip through ultrasound imaging combined with a magnetic needle-tracking system. Both groups receive the same injection technique and target the same anatomical site; however, the SNG uses a 26G × 60 mm needle, while the LNG uses a 25G × 100 mm needle. All other procedural variables remain consistent across groups.

During the procedure, performance-related indicators are recorded, including the number of needle insertions, total procedure time, confirmation of needle-tip arrival at the deep target layer (QPI space), and visualization of the hypoechoic spread during injectate delivery. All ultrasound images and needle-tracking log data are stored for subsequent analysis.

Immediately after the procedure, pain intensity (NRS), PRPPR, and ROM are reassessed. Additionally, the operator completes a usability questionnaire to evaluate needle visibility, tracking stability, handling comfort, confidence in target engagement, perceived procedural workload, and overall satisfaction. Any immediate adverse events occurring at this time point are documented in detail.

Follow-up assessments are conducted via telephone within 24 to 36 h after the procedure. During this contact, changes in pain intensity (NRS), PRPPR, functional status in daily activities, and the presence of any delayed adverse events are evaluated. All collected data are recorded using institution-specific Case Report Forms (CRFs) and included in the final analysis after de-identification. A summary of the study procedures is presented in Table 1.

**Table 1:** Summary of study procedures and assessment timeline.

Study procedure	Period		
	Screening*	Visit 1	F/U^†^
	-1D^‡^	Baseline (0)	~1D
Explanation of the study and obtaining consent	○		
Determination of inclusion/exclusion criteria	○		
Demographic survey^a^	○		
Medical history investigation^b^	○		
ODI (Baseline)^c^		○	
NRS (Pre-/Post-intervention, Day 1)^d^		○	○
PRPPR (Post-intervention, Day 1)^e^		○	○
ROM (Range of Motion) (Pre-/Post-intervention)^f^		○	
Intervention (Short needle vs. Long needle)		○	
Adverse event assessment^g^		○	○

*If the subject is deemed eligible after screening, the first intervention will be performed immediately after obtaining informed consent. ^†^A telephone-based follow-up will be conducted within 1 day (24–36 h) after Visit 1. ^‡^D indicates “Day.” ^a^Demographic survey: information on gender, age, year of birth, height, weight, and other baseline characteristics will be collected. ^b^Medical history: includes previous history of low back pain(LBP), hypertension, diabetes, osteoarthritis, sleep disorders, psychiatric history, current medications, smoking history, and alcohol consumption. ^c^ODI (Oswestry disability index): the ODI is a validated questionnaire assessing functional disability related to LBP. It consists of 10 items covering pain intensity and daily activities, and yields a score ranging from 0 to 100%, with higher scores indicating greater disability. In this study, ODI is administered only at baseline to evaluate initial functional status. ^d^NRS (numeric rating scale): pain intensity for LBP will be assessed on a 0–10 scale, where higher scores indicate more severe pain. ^e^PRPPR (patient-reported percentage pain reduction): subjects will be asked to report the perceived percentage of pain reduction compared with pre-intervention status, on a 0–100% scale, both immediately after treatment and at Day 1 follow-up. ^f^ROM (range of motion): lumbar ROM—including flexion, extension, lateral flexion, and rotation—will be assessed to evaluate functional changes. ^g^Adverse events: adverse events or unexpected symptoms occurring immediately after the procedure or during follow-up will be assessed and recorded.

The study protocol has been submitted for Institutional Review Board (IRB) approval. Ethical approval will be obtained prior to the initiation of participant enrollment. No participants have been recruited or exposed to the intervention at the time of manuscript submission.

### 2.2 Study registration

This study was registered with the Clinical Research Information Service (CRIS), Republic of Korea (KCT0011603; 11 February 2026).

### 2.3 Participants

The study will include adult participants diagnosed with acute LBP. Before enrollment, all potential participants will receive a comprehensive explanation of the study’s purpose, procedures, expected risks and benefits, data protection policies, and their rights and safety as research subjects. Voluntary participation will be confirmed through written informed consent obtained prior to any study-related procedures.

To ensure a clearly defined cohort and minimize variability in treatment response, only individuals experiencing symptoms for 7 days or less will be included, thereby limiting the sample to participants in the acute phase of LBP.

LBP is commonly classified based on symptom duration as acute (0–6 weeks), subacute (6–12 weeks), and chronic (more than 12 weeks) (3). Similarly, the U. S. National Institutes of Health (NIH) Task Force defines chronicity using a 3-month threshold (11). However, these broad temporal classifications (e.g., 6 or 12 weeks) may introduce substantial heterogeneity within clinical research populations, limiting the ability to accurately assess treatment effects.

Previous studies indicate that most improvements in pain and functional recovery among patients with acute LBP occur within the first 4 to 6 weeks after onset, followed by a markedly slower rate of natural recovery thereafter (3, 12, 13). This recovery trajectory suggests that patients within the first 1 to 2 weeks of symptom onset may represent a distinct clinical subgroup compared to those at 4 to 6 weeks, particularly regarding biological state and likelihood of spontaneous resolution. Moreover, beyond the early phase, greater variability in pain trajectory and prognosis has been reported, further complicating the evaluation of treatment effects. Accordingly, restricting enrollment to patients within the first 7 days of symptom onset may enhance sample homogeneity and improve the validity of early-phase intervention assessments.

Accordingly, the present study adopted a more stringent inclusion window, limiting participant eligibility to within 7 days of symptom onset (0–7 days) rather than the broader conventional acute definition of 0–6 weeks. This refinement aimed to ensure greater cohort homogeneity, minimize potential confounding from spontaneous recovery, and enable a more accurate evaluation of the therapeutic impact of early, targeted intervention in acute LBP.

#### 2.3.1 Setting

This multicenter study will take place across four Korean medicine clinics that provide primary outpatient care for musculoskeletal conditions. Participant enrollment and all research-related procedures will be conducted in the outpatient setting of each clinic.

The list of four Korean medicine clinics is as follows:

- Kangnyung Korean Medicine Clinic (Anyang, Gyeonggi Province, Republic of Korea).
- Gwanghwamun Kyung Hee Korean Medicine Clinic (Jongno-gu, Seoul Metropolitan City, Republic of Korea).
- Mullae Majubom Korean Medicine Clinic (Yeongdeungpo-gu, Seoul Metropolitan City, Republic of Korea).
- Mapo Hongik Korean Medicine Clinic (Mapo-gu, Seoul Metropolitan City, Republic of Korea).

The interventions will be performed exclusively by Korean medicine doctors who have undergone structured training in ultrasound-guided pharmacopuncture and who are active members of the Academy of Korean Medicine Clinical Anatomy, an organization specializing in ultrasound-based clinical anatomy research.

#### 2.3.2 Inclusion criteria

Participants will be included if they meet all of the following conditions:

1. Age between 20 and 64 years.
2. Acute LBP within 7 days of symptom onset, accompanied by lumbar pain and limited ROM.
3. LBP, with or without referred leg pain.
4. No prior treatment for the current episode of LBP (diagnostic imaging alone is permitted).
5. Ability to understand the study procedures and provide written informed consent.
6. Participants treated with ultrasound-guided pharmacopuncture using a long needle (25G × 100 mm) at Mapo Hongik Korean Medicine Clinic or Gwanghwamun KyungHee Korean Medicine Clinic, or using a short needle (26G × 60 mm) at Mullae Majubom Korean Medicine Clinic or Gangnyeong Korean Medicine Clinic.

#### 2.3.3 Exclusion criteria

Participants will be excluded if any of the following apply:

1. Participants who have received medication or related treatments for LBP or discomfort within the past 7 days, including treatment at medical (Western medicine) or Korean medicine institutions.
2. Participants with a history of lumbar spine surgery within the past 3 months.
3. Participants who developed lumbar sprain due to trauma (e.g., traffic accident, fall, or contusion) within the past 3 months.
4. Female Participants who are currently pregnant or who have given birth within the past 6 months.
5. Participants with renal or endocrine disorders accompanied by symptoms such as edema.
6. Participants currently taking antithrombotic agents (including anticoagulants or antiplatelet medications).
7. Participants with cognitive impairment that makes it difficult to understand the study procedures or provide voluntary informed consent.
8. Participants currently taking psychiatric medications for conditions such as depression.
9. Participants deemed unsuitable for participation at the discretion of the investigator (e.g., refusal to follow instructions, safety concerns).

#### 2.3.4 Dropout criteria

This study involves a single intervention (Visit 1), and participants who complete this procedure will not be classified as dropouts. The follow-up assessment conducted 24–36 h later serves as an additional evaluation; therefore, lack of response to this assessment does not constitute withdrawal, and participation will still be considered valid. Dropout criteria apply only under the following circumstances:

1. *Withdrawal of consent*: If the participant or their legal representative requests termination of study participation or withdraws informed consent at any time.
2. *Inability to perform the procedure*: If the participant’s medical or physical condition changes before the intervention, and the investigator determines that performing ultrasound-guided pharmacopuncture is no longer safe.
3. *Unexpected medical issues following the procedure*: If a significant adverse reaction or medical complication occurs during or after the intervention, and continued participation is considered inappropriate.
4. *Investigator decision*: If the investigator determines that continued participation jeopardizes participant safety, compromises data integrity, or undermines the ethical conduct of the study.

Non-response to the 24–36-h follow-up assessment alone does not constitute dropout; such missing data will be treated as simple missing values during analysis.

#### 2.3.5 Early termination criteria

The study team may terminate the trial early if continuing the prospective comparative observational study is deemed inappropriate. Early termination may be implemented to ensure participant safety, maintain data integrity, and uphold ethical and administrative feasibility. The following conditions may justify early termination:

1. *Safety-related reasons*: When a serious safety concern related to the procedure or study process arises, and participant protection can no longer be guaranteed.
2. *Protocol or ethical violations*: When the study site or research team significantly violates the approved protocol, IRB requirements, applicable regulations, or ethical standards, making the continuation of the study inappropriate.
3. *Administrative or operational reasons*: When the continuation of a study becomes substantially infeasible due to institutional administrative decisions, operational challenges, inability to secure necessary resources, or similar limitations.

In the event of early termination, the principal investigator will compile all data collected up to that point, including CRFs and related documents, and submit a summary report to the IRB and the institution. All study documents and collected data will be retained or disposed of in accordance with institutional policies and IRB guidelines.

#### 2.3.6 Management of early termination and dropout

In cases of early termination or participant dropout during the study, the investigator will document the reason and timing of withdrawal in both the CRF and the participant’s medical record. If the withdrawal is suspected to be related to an adverse event, follow-up contact will be made to assess the participant’s clinical status. When an adverse event is confirmed, appropriate medical care will be provided in accordance with the clinical guidelines of each participating institution.

If the study is terminated prematurely at either the institutional or overall study level, the principal investigator will compile all collected study materials—including participant records and study documents—up to the point of termination and report them to the IRB and the relevant institution. All data and materials will then be stored or disposed of in accordance with institutional policies and IRB requirements.

Participants who discontinue the study will not be re-enrolled. The inclusion of data collected prior to withdrawal in the final analysis will be determined according to the study protocol and the predefined statistical analysis plan. Since the follow-up assessment at 24–36 h is considered an additional exploratory endpoint, the absence of follow-up data alone will not be classified as dropout. Non-responses at this time point will be treated as missing data and handled accordingly in the analysis.

#### 2.3.7 Participant recruitment plan

Participant recruitment will take place naturally within the outpatient clinical settings of the participating institutions. Information sheets describing the study will be posted in each clinic to enable patients with acute LBP to voluntarily inquire about or express interest in participation. Additionally, clinicians may introduce the study to eligible patients during routine consultations if they meet the inclusion criteria.

Supplementary recruitment strategies, such as utilizing community bulletin boards or online platforms (e.g., institutional websites or blogs), may also be employed to enhance awareness of the study. All recruitment procedures will strictly adhere to the methods approved by the IRB.

#### 2.3.8 Registration of participants

Individuals expressing interest in participating will be provided with detailed information about the study’s objectives, procedures, potential benefits and risks, and data privacy protections. The registration process will begin only after participants voluntarily sign the written informed consent form.

Following consent, eligibility will be assessed based on predefined inclusion and exclusion criteria. Participants who meet all criteria will be formally enrolled in the study, and baseline assessments and the intervention will be conducted during the first visit (Visit 1). All procedure-related clinical information and evaluation outcomes will be systematically recorded using a standardized CRF.

### 2.4 Intervention

All procedures in this study will be performed under real-time ultrasound guidance using a portable ultrasound system equipped with a magnetic needle-tracking function (AcuViz Pocket, FCU Corp., Korea). This device incorporates a magnetic sensor system that detects the position and trajectory of magnetized needles and displays them in real time on the ultrasound screen via a Needle Guiding System (NGS), thereby enhancing needle visibility during insertion.

Two types of magnetized needles will be used depending on group allocation. The Acu-tracking needle (26G × 60 mm; YONG CHANG Co., LTD., Korea) will be used for procedures in the SNG, while the Ez-tracking needle (25G × 100 mm; YONG CHANG Co., LTD., Korea) will be used for the LNG.

The pharmacopuncture solution used in this study is a salmon (Oncorhynchus spp.)-derived PDRN formulation. A 2 mL vial of PDRN pharmacopuncture solution (Yeona Pharmacopuncture; AJ External Herbal Dispensing Facility, Seoul, Republic of Korea), an MOHW-certified compounding facility, will be used for all procedures. PDRN consists of purified DNA polymer fragments (Polynucleotides) and has been reported to exert anti-inflammatory effects, promote tissue regeneration, improve microcirculation, and reduce pain (14). Accordingly, PDRN-based pharmacopuncture has been clinically utilized for lumbar pain, spinal stenosis, and radicular symptoms (15–17).

For administration, the solution will be aspirated into a 3 cc disposable syringe (Sungshim Medical Co., Republic of Korea) using an attached sterile needle. The needle will then be replaced with either the Acu-tracking needle or the Ez-tracking needle, according to group assignment, before injection.

#### 2.4.1 Ultrasound-guided interfascial injection procedure at the QPI space

**Figure 3:**
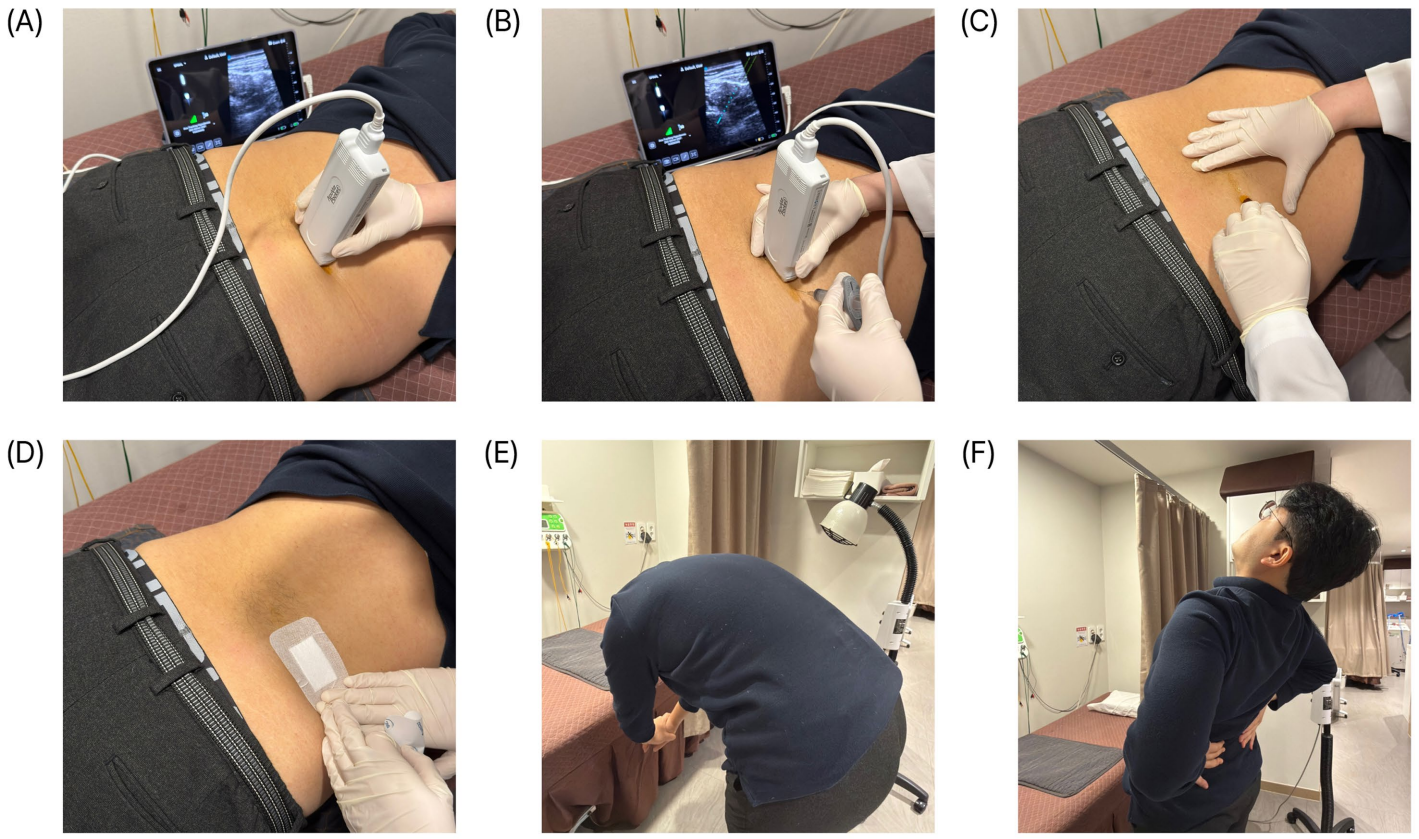
Ultrasound-guided pharmacopuncture procedure and post-treatment process in the QPI space. **(A)** Identification of the transverse process of the third lumbar vertebra (L3) and the iliocostalis, psoas major, and quadratus lumborum muscles using ultrasound imaging. **(B)** After skin disinfection at the needle insertion site, the needle is advanced using an in-plane ultrasound-guided approach, and pharmacopuncture solution is injected into the QPI interfascial space. **(C,D)** Upon completion of the injection, the needle is withdrawn, hemostasis is performed if bleeding occurs, and a sterile dressing is applied. **(E,F)** Mild lumbar flexion, extension, and rotation are performed to assess symptom changes and to monitor for any immediate adverse reactions. The model depicted in this figure is one of the investigators participating in this study and provided prior consent for the use of the photograph in the publication.

1. The L3 spinous process is initially identified using an ultrasound device (AcuViz Pocket).
2. The probe is then rotated 90 degrees to obtain a transverse view and visualize the transverse process of L3.
3. The probe is moved slightly cranially until the PM, previously obscured by the transverse process, becomes visible.
4. The probe is adjusted until the medial border of the QL aligns approximately with the lateral border of the IC muscle.
5. After identifying the IC, QL, and PM muscles, the probe is stabilized once a peristaltic bowel movement is observed lateral to the PM, indicating the appropriate depth and confirming the anatomical landmarks.
6. The skin over the planned needle entry site is thoroughly disinfected over a sufficiently wide area.
7. Using an in-plane approach, the magnetic pharmacopuncture needle is advanced toward the medial border of the QL muscle and positioned within the interfascial plane. The injectate is then slowly administered to facilitate natural spread toward the PM fascia.
8. During the procedure, the patient may experience transient neural sensations, such as radiating tingling, warmth, numbness, or a relieving sensation in the lower limb.
9. After completing the injection, the needle is withdrawn, and hemostasis is achieved if bleeding occurs, followed by the application of a sterile dressing.
10. The patient is instructed to stand and perform gentle lumbar flexion, extension, and rotation to assess any changes in symptoms or adverse reactions (Figure 3).

### 2.5 Outcomes

#### 2.5.1 Primary outcome

The primary outcome of this study is the change in Numeric Rating Scale (NRS) scores for LBP immediately following ultrasound-guided pharmacopuncture.

The NRS score recorded immediately before the procedure will serve as the baseline, and a second NRS assessment will be conducted immediately after the intervention (QPI-guided PDRN pharmacopuncture). The difference between the baseline and post-procedure scores will be calculated to determine the extent of symptom improvement. A between-group comparison of NRS changes (SNG versus LNG) will be used to evaluate whether needle length influences the magnitude of acute pain reduction.

#### 2.5.2 Secondary outcomes

##### 2.5.2.1 Change in lumbar ROM

Lumbar ROM (flexion, extension, lateral flexion, and rotation) will be assessed immediately before the procedure and reassessed immediately afterward. The change from baseline (ΔROM) will be calculated to evaluate the short-term functional improvement associated with needle length. Between-group differences in ΔROM will be analyzed to determine whether long needles provide greater mobility gains compared to short needles.

Lumbar ROM will be measured using a standard goniometer. Flexion and extension will be assessed in a standing position, with the axis placed lateral to the lumbosacral joint, the stationary arm aligned vertically, and the moving arm aligned with the lateral trunk line. Lateral flexion will be measured from the posterior aspect, with the axis positioned over the midline of the lumbosacral junction, the stationary arm parallel to the line connecting the posterior superior iliac spines (PSIS), and the moving arm directed along the spinous processes toward C7. Rotation will be measured in a seated position, with the axis located at the cranial midline, the stationary arm parallel to the floor, and the moving arm directed toward the contralateral acromion (18).

##### 2.5.2.2 Functional disability assessed using the ODI

The ODI is a standardized, self-reported questionnaire designed to assess functional disability related to LBP. It comprises 10 items, and the final score is expressed as a percentage ranging from 0 to 100%, with higher scores indicating greater disability. In this study, the baseline ODI score will be used as a descriptive measure of the participants’ functional limitations (19).

##### 2.5.2.3 Patient-reported percentage pain reduction, PRPPR

PRPPR is a subjective metric in which participants rate their pre-procedure pain intensity as 100%, then report the percentage by which their pain has decreased after the procedure on a 0–100% scale. In pain intervention research, it has been suggested that evaluating the perceived percentage reduction—not just the absolute numerical change (e.g., NRS difference)—may provide a more sensitive measure of clinical improvement, especially when assessing early or immediate treatment response (20–22). Based on this rationale, PRPPR was selected as a secondary outcome measure in this study to capture the immediate analgesic effect following a single session of ultrasound-guided pharmacopuncture.

As this study is a prospective, comparative, observational trial conducted in a real-world clinical setting, complete assessor blinding based on needle type (Short vs. Long) is not feasible. Therefore, outcome assessors will evaluate participants in an unblinded state, with access to group allocation information.

Although this approach may introduce potential measurement bias, several strategies have been implemented to minimize its impact. All participating centers will adhere to a standardized assessment protocol, and outcome assessors will receive uniform training prior to the study’s initiation. Furthermore, all outcome measures—including NRS, ROM, ODI, and PRPPR—will be recorded consistently according to predefined operational definitions.

Despite these procedures, residual bias inherent in non-blinded assessments cannot be entirely eliminated. This limitation should be considered when interpreting the study findings.

### 2.6 Sample size and statistical analysis

#### 2.6.1 Sample size calculation

Since no prior study has been conducted under conditions identical to the present trial, the effect size for this study was estimated based on comparable interventions and studies reporting changes in NRS or VAS as primary outcomes. In a double-blind randomized controlled trial evaluating pharmacopuncture in patients with chronic LBP, the mean reduction in pain after 3 weeks was 2.74 points in the treatment group and 1.76 points in the control group, yielding an estimated between-group difference of approximately 1.0 point and a Cohen’s d of approximately 0.56 (23). Similarly, a systematic review of acupuncture for acute LBP reported a mean difference of −1.75 points on the VAS compared with controls (95% CI: −2.39 to −1.12) (24), indicating a meaningful short-term analgesic benefit. Additionally, a single-arm study conducted in Japan evaluating ultrasound-guided hydrodissection demonstrated a reduction of more than 4 points on the VAS immediately after the procedure (25), suggesting that ultrasound-guided interventions may provide rapid short-term pain relief.

Additionally, although the Minimum Clinically Important Change (MCIC) for acute LBP has been reported to be approximately 3.5 points on the NRS scale (26), such thresholds primarily apply to single-arm studies evaluating absolute symptom improvement. In comparative trial designs, such as the present study, the full magnitude of the MCIC is not required to determine a meaningful treatment difference. Instead, between-group differences of approximately 1 point—similar to those reported in previous randomized controlled trials—may still represent a clinically relevant effect when assessing comparative therapeutic outcomes.

Based on the literature reviewed, the effect size for this study was set at Cohen’s d = 0.56. Using a two-sample *t*-test with a significance level of 0.05 and a statistical power of 80%, the minimum required sample size was calculated to be 104 participants (52 per group). Allowing for an anticipated dropout rate of approximately 10%, the final target sample size was set at 120 participants, with 60 allocated to the short-needle group and 60 to the long-needle group.

#### 2.6.2 Statistical analysis

All statistical analyses will be performed using R software (version 4. X. X; R Foundation for Statistical Computing, Vienna, Austria). All tests will be two-tailed, with statistical significance defined as *p* < 0.05. Continuous variables will be summarized as mean ± standard deviation or median with Interquartile Range (IQR), depending on the data distribution. Categorical variables will be presented as frequencies and percentages.

For the primary outcome, changes in NRS scores will be analyzed according to the data distribution. Between-group comparisons will be performed using an independent *t*-test if the normality assumption is satisfied; otherwise, the Wilcoxon rank-sum test will be used. Within-group changes will be assessed using a paired *t*-test or the Wilcoxon signed-rank test, depending on whether the data meet normality assumptions.

For the secondary outcomes, changes in lumbar ROM from baseline will be analyzed using the same statistical methods applied to the primary outcome. Between-group comparisons will be conducted using an independent *t*-test or the Wilcoxon rank-sum test, while within-group comparisons will be analyzed using a paired *t*-test or the Wilcoxon signed-rank test, depending on data normality.

The ODI will be categorized into predefined functional disability levels, and differences in distribution between groups will be analyzed using the Chi-square test or Fisher’s exact test, as appropriate.

PRPPR will be assessed immediately after treatment and again at 24–36 h post-intervention. Between-group comparisons will be conducted using an independent *t*-test or the Wilcoxon rank-sum test, while within-group comparisons will be analyzed using a paired *t*-test or the Wilcoxon signed-rank test, depending on the data characteristics.

In addition, procedural characteristics and usability-related variables associated with needle length—including deep-layer reach success rate, number of insertions, procedure time, and operator usability score—will be summarized using descriptive statistics. When appropriate, exploratory between-group comparisons will be conducted using the Mann–Whitney U test or the Chi-square test. These analyses will serve as supportive reference analyses and will not directly influence the interpretation of the primary clinical outcomes.

If significant baseline differences are identified between the two groups and these variables are considered potentially related to the outcome measures, they will be treated as possible confounding factors. In such cases, subgroup analyses or multivariate analyses (e.g., ANCOVA or multiple regression models) will be conducted. Adjusted effect sizes with corresponding 95% confidence intervals will be reported to improve the interpretability and accuracy of the findings.

### 2.7 Data collecting, processing, and monitoring

All procedures for data collection and management will be conducted in accordance with the clinical research Standard Operating Procedures (SOPs) of each participating institution. For any procedures not specified in the institutional SOPs, the International Council for Harmonisation–Good Clinical Practice (ICH-GCP) guidelines will be followed. Study data will be entered into the CRF or electronic CRF (eCRF) immediately after the intervention. If any data are missing, the reason for the omission will be documented.

Collected data will be reviewed against source documents to ensure accuracy and consistency. All related study documentation will be archived to allow verification upon request after study completion. Documents containing personal identifiers will be securely stored in a locked facility for a minimum of 3 years, in accordance with institutional clinical research regulations. Electronic personal data will be encrypted and stored on secure systems.

Data monitoring will be conducted by designated research personnel at each site. The monitoring process will include routine verification of data entry accuracy, consistency, completeness, and compliance with the approved study protocol.

### 2.8 Ethical considerations

#### 2.8.1 Ethical approval

This study protocol has been reviewed and approved by the Public IRB designated by the Ministry of Health and Welfare, Republic of Korea (approval number: P01-202602-01-019). The study will be conducted in accordance with the ethical principles outlined in the Declaration of Helsinki and relevant national regulations governing clinical research involving human participants.

#### 2.8.2 Informed consent

Prior to enrollment, all participants will receive a comprehensive explanation of the study objectives, procedures, potential risks and benefits, and data protection measures. Written informed consent will be obtained from all participants who voluntarily agree to participate after receiving and understanding this information. The informed consent process will be conducted in accordance with the approved IRB documents and the institution’s SOPs.

#### 2.8.3 Management and compensation adverse events

In the event of a Serious Adverse Event(SAE) or medical emergency occurring during the study, participants will receive immediate and appropriate medical care in accordance with institutional clinical protocols. If an adverse event is determined to be related to the study intervention, compensation or necessary medical support will be provided in compliance with institutional policies and applicable regulations. All SAEs will be reported to the IRB in a timely manner, in accordance with established reporting requirements.

### 2.9 Adverse events

The pharmacopuncture solution used in this study contains salmon-derived PDRN, a substance reported to exert anti-inflammatory and tissue-regenerative effects in various musculoskeletal conditions (16). Although PDRN pharmacopuncture is widely utilized in primary care settings and is generally considered safe, mild adverse reactions such as bruising, localized discomfort, or transient fatigue may occur; therefore, these possibilities will be explained to participants in advance.

The magnetic needle used in this study is inserted into deep tissue layers under ultrasound guidance. Similar to other acupuncture-based procedures, potential adverse reactions may include localized pain or tenderness at the insertion site, transient paresthesia (such as tingling, prickling, or burning sensations), minor bleeding or hematoma, and, less frequently, skin hypersensitivity, infection, dizziness, syncope, nausea, or headache.

To ensure participant safety, all adverse events will be assessed immediately after the procedure and reassessed 24–36 h later via telephone or mobile follow-up. If an adverse event occurs, investigators will promptly report it to the principal investigator. In cases where a SAE is suspected, appropriate clinical care will be provided immediately in accordance with institutional medical protocols.

All adverse events will be documented in the CRF, including symptom characteristics, onset date, duration, severity, and potential causal relationship to the intervention. SAEs will be reported to the IRB according to institutional reporting requirements. Upon study completion, adverse event data will be summarized descriptively by frequency, type, and incidence within each study group for safety evaluation.

## 3 Discussion and conclusion

This study is a prospective, multicenter observational protocol designed to evaluate the feasibility and short-term clinical response of ultrasound-guided pharmacopuncture targeting the interfascial QPI space in patients with acute LBP in a primary care setting.

Recently, interest in ultrasound-guided acupuncture and pharmacopuncture has grown within Korean musculoskeletal clinical practice, and related studies have gradually emerged (27, 28). However, significant heterogeneity in study design, target tissue layers, pharmacological formulations, and outcome measures has hindered the establishment of consistent clinical standards (29–31). Notably, prospective research investigating interfascial QPI-space approaches in patients with acute LBP remains extremely limited, with existing literature largely restricted to retrospective case reports (7). Accordingly, this study provides preliminary feasibility-level clinical evidence and serves as an exploratory step toward evaluating the therapeutic and procedural relevance of this technique.

A key strength of this study is its design, which directly compares how differences in needle length (Short versus Long) affect both procedural performance and short-term clinical response within a single standardized intervention—ultrasound-guided pharmacopuncture targeting the interfascial QPI space. Unlike previous studies that primarily reported clinical outcomes or provided case-based descriptions using a single needle length, this study systematically collected procedural data, enabling evaluation of technical factors such as needle-tip visibility during deep advancement, accessibility of the target layer across different body habitus, operator handling characteristics, and perceived procedural difficulty. This approach extends beyond a simple comparison of clinical efficacy and enables a more comprehensive assessment of the reproducibility and practical feasibility of deep interfascial access, thereby distinguishing this study from prior literature.

The interfascial plane of the lumbar region can present varying levels of technical difficulty depending on factors such as body habitus (including BMI and muscle thickness), probe–needle alignment, acoustic properties, and anatomical variations. Anatomical and imaging studies indicate that the target layer is typically located at a depth of approximately 4–6 cm or more from the skin surface (32). Given this depth, conventional 50–60 mm needles may provide limited access or an insufficient safety margin in patients with increased soft tissue thickness. In contrast, anesthesia literature shows that 80–100 mm block needles are routinely used for similarly deep interfascial approaches, such as QL or Erector Spinae Plane (ESP) blocks (33–37). However, within Korean medicine clinical practice, systematic validation of long-needle use—particularly regarding safety, reproducibility, and ultrasound visibility—has been limited, resulting in selective or restricted adoption in real-world settings. Accordingly, the design of the present study holds both methodological and clinical relevance by addressing this gap and evaluating the feasibility of long-needle application in ultrasound-guided interfascial pharmacopuncture.

This study has several methodological strengths, including its prospective design, needle-length comparison framework, multicenter real-world data collection, and incorporation of practitioner usability assessments. Notably, the study was designed to collect not only clinical response outcomes but also procedural variables such as needle handling experience, perceived technical difficulty, and confidence in reaching the target plane. This approach enables exploration of the relationship between therapeutic outcomes and procedural experience. Furthermore, conducting the study in a real-world outpatient clinical setting enhances the applicability of the findings, as it reflects variability in patient characteristics, procedural accessibility, and the operator learning curve. These features contribute to the study’s potential value for future clinical implementation and the development of educational frameworks.

Despite these strengths, this study has several inherent limitations due to its exploratory observational design. First, the site-based allocation approach may introduce selection bias. Second, because the intervention was conducted as a single session with short-term follow-up (24–36 h), the study design does not permit evaluation of sustained or cumulative treatment effects. Third, not all participants underwent imaging-based diagnostic confirmation, which may lead to diagnostic heterogeneity and should be taken into account when interpreting the findings.

Future research should include randomized controlled trials to verify the independent effects of needle length and NGS application, as well as quantitative imaging-based assessments of needle tip visibility and tracking accuracy. Additionally, studies incorporating repeated interventions, long-term follow-up, and subgroup analyses based on BMI, muscle thickness, or pain phenotype may further clarify patient-specific treatment responses. Finally, cost-effectiveness analyses would help determine the practical value of ultrasound-guided interfascial pharmacopuncture in primary care settings and contribute to establishing clearer clinical indications and standardized application criteria for the QPI-guided approach.

## Glossary

LBP: Low back pain
QL: Quadratus Lumborum
PM: Psoas Major
IC: Iliocostalis
QPI space: Interfascial plane formed by the Quadratus Lumborum, Psoas Major, and Iliocostalis muscles
PDRN: Polydeoxyribonucleotide
SNG: Short Needle Group
LNG: Long Needle Group
NRS: Numerical Rating Scale
ROM: Range of Motion
ODI: Oswestry Disability Index
PRPPR: Patient-reported Percentage Pain Reduction
CRFs: Case Report Forms
IRB: Institutional Review Board
MCIC: Minimum Clinically Important Change
SOPs: Standard Operating Procedures
ICH-GCP: International Council for Harmonisation–Good Clinical Practice
eCRF: Electronic CRF
SAE: Serious Adverse Event
ESP: Erector Spinae Plane
